# Effect of two yoga-based relaxation techniques on memory scores and state anxiety

**DOI:** 10.1186/1751-0759-3-8

**Published:** 2009-08-13

**Authors:** Pailoor Subramanya, Shirley Telles

**Affiliations:** 1 Indian Council of Medical Research Center for Advanced Research in Yoga and Neurophysiology, SVYASA, Bangalore, India

## Abstract

**Background:**

A yoga practice involving cycles of yoga postures and supine rest (called cyclic meditation) was previously shown to improve performance in attention tasks more than relaxation in the corpse posture (*shavasana*). This was ascribed to reduced anxiety, though this was not assessed.

**Methods:**

In fifty-seven male volunteers (group average age ± S.D., 26.6 ± 4.5 years) the immediate effect of two yoga relaxation techniques was studied on memory and state anxiety. All participants were assessed before and after (i) Cyclic meditation (CM) practiced for 22:30 minutes on one day and (ii) an equal duration of Supine rest (SR) or the corpse posture (*shavasana*), on another day. Sections of the Wechsler memory scale (WMS) were used to assess; (i) attention and concentration (digit span forward and backward), and (ii) associate learning. State anxiety was assessed using Spielberger's State-Trait Anxiety Inventory (STAI).

**Results:**

There was a significant improvement in the scores of all sections of the WMS studied after both CM and SR, but, the magnitude of change was more after CM compared to after SR. The state anxiety scores decreased after both CM and SR, with a greater magnitude of decrease after CM. There was no correlation between percentage change in memory scores and state anxiety for either session.

**Conclusion:**

A cyclical combination of yoga postures and supine rest in CM improved memory scores immediately after the practice and decreased state anxiety more than rest in a classical yoga relaxation posture (*shavasana*).

## Findings

Yoga includes practices such as physical postures, regulated breathing, and meditation, among other techniques [1]. Meditation practice reduces stress and increases calmness [2], but many novices find it difficult to practice meditation initially [3]. In fact, meditation is the seventh of eight steps traditionally described [Patanjali, *circa *900 B.C.] [1]. Some people find it easier to begin by practicing yoga postures. Based on this a 'moving meditation' called cyclic meditation was evolved which has cycles of yoga postures alternating with guided relaxation while supine [3]. Cyclic meditation practice improved the performance in a P300 event related potential task [4] and also improved the performance in a letter cancellation task [5]. Both tasks require selective attention and concentration. The benefits were ascribed to possible stress reducing effects of cyclic meditation, as the practice reduces physiological [6,7] and cortical [8] arousal. However the effects of cyclic meditation on state anxiety have not been assessed.

In the present study cyclic meditation was compared to an equal duration of supine rest in the corpse posture (*shavasana*), as both are supposed to increase relaxation. Hence, the present study was designed to assess the effects of cyclic meditation and *shavasana *on state anxiety and the performance in memory tasks, to see whether they would change after the practices.

There were 57 male participants, aged between 18 and 40 years (group average age ± S.D., 26.6 ± 4.5 years), all with normal health and not on medication. They were residing at a yoga center. All of them had a minimum of 15 years of education and could understand the tasks. Their experience of cyclic meditation and of relaxation in the corpse posture (*shavasana*) was between 6 and 48 months (group average 20.1 ± 14.9 months). The study had been explained to the participants, whose signed informed consent was taken. The Institutional Ethics committee approval was obtained.

All participants were assessed before and after two practice sessions, viz., cyclic meditation (CM) and supine rest (SR) in *shavasana*. At random twenty-nine participants had CM on the first day, and SR the next day. The remaining participants had the reverse schedule. The time of day was kept constant for both sessions of an individual. Sessions were 22:30 minutes in duration.

Memory tasks were selected from the Wechsler memory scale which has been standardized for use in an Indian population. The following sections were selected (i) digit span forward and backward, and (ii) verbal paired associate learning (easy and hard), with 10 items each. The verbal paired associate learning task involved the presentation of ten pairs of unrelated words as three trials. After the three trials the examinee was presented with the first word in each pair and he or she was asked to provide the second word. Out of the ten pairs, six pairs were semantically easy to remember (e.g., table-chair). Where such associations existed, it was described as associate learning, easy. Where there were no such associations the task was described as associate learning, hard. There were six pairs for the easy task and four pairs for the hard task. Each correct answer was scored as '1' (for digit span forward or backward), while for associate learning, each easy answer was scored as '1' and difficult or hard answer as '2'. This was based on the conventional scoring for Wechsler memory scale [9]. Parallel worksheets were prepared, changing the digits and words to eliminate serial testing artifacts when retesting [10].

State anxiety was assessed using the Spielberger's State-Trait Anxiety Inventory at the beginning and end of the CM and SR sessions, after the memory tasks.

Test sheets were blind scored by a person who was unaware about the participant's practice session or whether the assessments were before or after a practice session.

During cyclic meditation participants kept their eyes closed and followed pre-recorded instructions. The emphasis was on carrying out the practice slowly, with awareness and relaxation. The practice has been detailed in earlier reports [4,6], but is described here in brief. The practice begins with isometric contraction of the muscles of the body while supine (1:00 min), followed by a standing posture (2:20 min), and two side bending postures (3:50 min). This is followed once more by a standing posture (2:20 min), a forward bending posture (1:30 min) and a backward bending posture (1:30 min). These yoga postures are followed by 10:00 min of guided relaxation while supine, with instructions to relax different parts of the body while being aware of them.

Relaxation in the corpse posture (*shavasana*) or supine rest was for the same duration, i.e., 22:30 min. This is a classic yoga posture, intended for relaxation [11]. Here, participants lie flat on the ground with their legs apart, arms away from the sides of the body, palms facing upwards and the eyes closed. During the training, participants had been instructed to attempt to remain relaxed while being aware of body sensations during *shavasana*.

Data were analyzed using SPSS (Version 16.0). There were separate repeated measures analyses of variance (ANOVAs) for each of the assessments, with two Within Subjects factors [i.e., States (before, after) and Sessions (CM, SR)]. *Post-hoc *analysis was with Bonferroni adjustment, comparing after with before values. The percentage change in each of the memory tasks {(where percentage change was [(After-value/Before-value*100)-100]} was tested for correlation with state anxiety using the Pearson correlation test.

Digit span forward scores differed significantly between Sessions (F = 4.1, p = 0.048), and between States (F = 286.4, p < 0.001), with a significant interaction between them (F = 13.4, p < 0.001). Digit span backward scores also differed between Sessions (F = 15.7, p < 0.001), and States (F = 124.4, p < 0.001) with a significant interaction between them (F = 37.9, p < 0.001). Similarly, associate learning, easy scores differed significantly between Sessions (F = 16.5, p < 0.001), and States (F = 237.9, p < 0.001), with the interaction between the two being significant (F = 37.1, p < 0.001). Also, for associate learning, hard, the scores differed significantly between Sessions (F = 16.4, p < 0.001) and States (F = 268.5, p < 0.001), with a significant interaction between them (F = 94.4, p < 0.001).

The state anxiety scores also differed significantly between Sessions (F = 54.9, p < 0.001), and States (F = 175.5, p < 0.001), with a significant interaction between them (F = 178.8, p < 0.001).

A significant interaction between factors, suggests that the two factors are not independent of each other, or one factor may be modified by the other factor.

**Figure 1:**
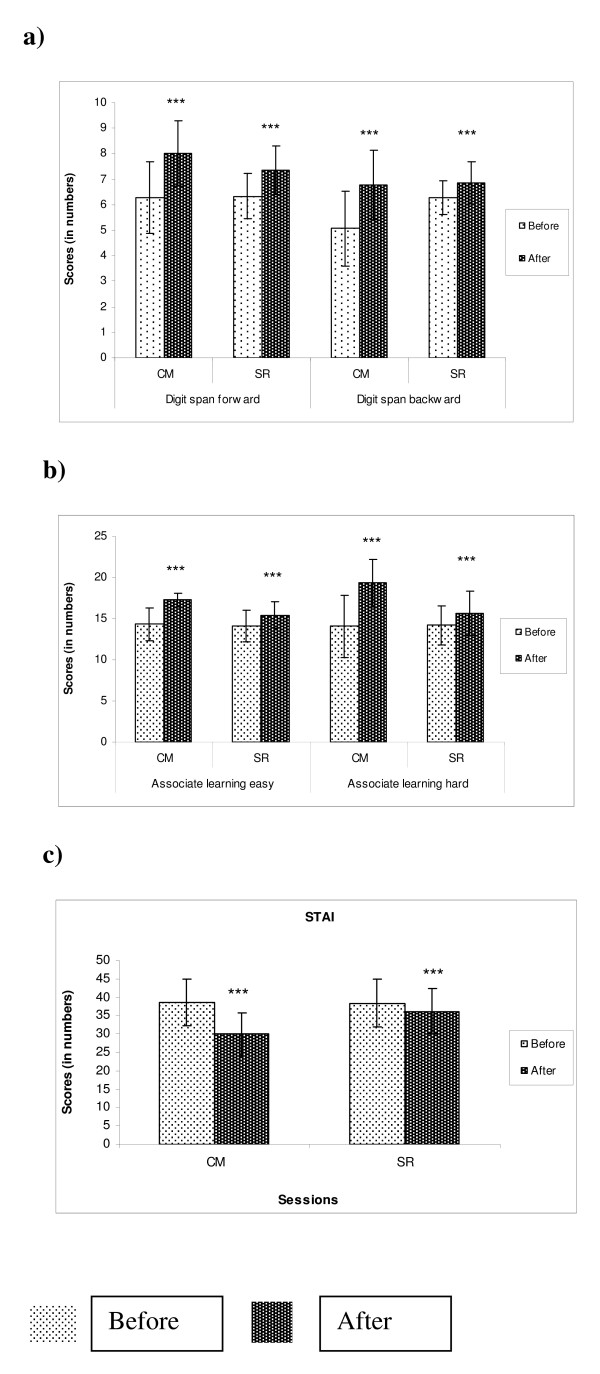
**Change in scores (mean ± SD) of (a) Digit-span forward and backward, (b) Associate learning, easy and hard, and (c) State anxiety, before and after CM and SR**. *** p < 0.001, after compared to before (*post-hoc *analysis).

*Post-hoc *analyses showed that following both CM and SR there was a significant increase in digit span forward scores (p < 0.001 in both cases), digit span backward scores (p < 0.001 in both cases), associate learning, easy (p < 0.001 in both cases) and associate learning, hard (p < 0.001 in both cases). State anxiety scores decreased after both CM and SR (p < 0.001 in both cases). All values for *post-hoc *analyses were Bonferroni adjusted (Figure 1).

The increase in scores for the digit span and associate learning tasks following CM was greater [digit span forward (27.7 percent), backward (33.5 percent), associate learning, easy (20.7 percent), and associate learning hard (37.7 percent)] than the increase following SR [digit span forward (16.1 percent), backward (9.2 percent), associate learning, easy (9.4 percent), and associate learning, hard (10.6 percent)]. Also, there was a greater magnitude of decrease in state anxiety after CM (22.4 percent) compared to after SR (5.6 percent). The digit span tests assess attention, concentration and primary working memory [12]. Earlier studies have shown that CM practice increases selective attention more than an equal duration of supine rest [4,5]. The present results suggest that primary working memory also improves with CM practice. Verbal paired associate learning assesses integration of information and episodic memory. The present results suggest an improvement in these aspects of memory after both CM and SR, with a greater magnitude of increase after CM.

Cyclic meditation involves movement, and such practices (another example being Tai-Chi-Qui-Gong) have been described as 'moving meditations' [13]. These techniques are described as meditations because during these practices practitioners ideally assume a meditative state of mind. This is characterized by interoception, awareness of body sensations, and relaxation [14]. Hence though these moving meditations differ from the classic description of meditation, in which the practitioners remain seated, keeping as still as possible, the mental state in both practices is supposed to be comparable.

The present results suggest that movement as a part of cyclic meditation may actually facilitate performance in attention and memory tasks more than an equal duration of time in a conventional relaxation posture (*shavasana*). A major drawback of the study is that participants were residing at the yoga center, and though they were not specifically told about the previous studies, they had access to them and this could have influenced their performance and hence the outcome. An attempt would be made to conduct the assessments on participants who are trained in CM but have no access to the findings reported earlier.

## Conflict of interests

PS and ST have no conflicts of interest in relation to this article.

## Authors' contributions

PS carried out the assessments, the data analysis and participated in compiling the manuscript. ST conceived and designed the study, and compiled the manuscript. Both authors read and approved the final manuscript.
